# Magneto-structural coupling in $Ni_xZn_{1-x}Cr_2O_4$

**DOI:** 10.1186/s40064-015-1224-z

**Published:** 2015-09-02

**Authors:** A. Khan, H. Kaneko, H. Suzuki, S. Naher, M. H. Ahsan, M. A. Islam, M. A. Basith, H. M. B. Alam, D. K. Saha

**Affiliations:** Shahjalal University of Science and Technology, Sylhet, 3114 Bangladesh; Graduate School of Natural Science and Technology, Kanazawa University, Kakuma-machi, Kanazawa, 920-1192 Japan; Bangladesh University of Engineering and Technology, Dhaka, 1000 Bangladesh; Atomic Energy Commission, Agargaon, Sher-e-Bangla Nagor, Dhaka, 1207 Bangladesh

**Keywords:** Magneto-structural coupling, Particle size, Low temperature X-ray diffraction

## Abstract

$ZnCr_2O_4$ compound is well Known to show the frustration of the spin structure. At 12 K, $ZnCr_2O_4$ distorts to break symmetry of the degenerated frustrated spin states by the spin-Peierls-like phase transition, accompanying with the antiferromagnetic ordering. On the other hand, $NiCr_2O_4$ undergoes a Jahn–Teller phase transition at a temperature of 310 K, differing from the low temperature ferrimagnetic transition temperature $T_c$ of about 60 K. It is also reported that $NiCr_2O_4$ shows another magnetic phase transition at about 30 K. These two phase transitions accompanying with the lattice change can be understood by the magneto-elastic interactions. Two interactions, the Jahn–Teller interaction and the spin-Peierls-like interaction are co-exist in $Ni_xZn_{1-x}Cr_2O_4$ system. In this report the $Ni_xZn_{1-x}Cr_2O_4$ compounds with x = 0.8, 0.6 and 1 are investigated by the X-ray diffraction measurements. From these measurements the crystal structures are determined. The full width at half maximum and integrated intensity give the fruitful information for magnetic elastic interactions.

## Background

Strong magneto-structural coupling in spinel structure results in rich behaviors such as the co-operative Jahn–Teller (JT) distortion and the spin liquid state. To study these effects, $Ni_xZn_{1-x}Cr_2O_4$ system has been investigated here. These compounds are normal cubic spinel with a space group $Fd\bar{3}m$ above 310 K for $NiCr_2O_4$ and at above 12 K for $ZnCr_2O_4$. In the normal spinel structure, such as $NiCr_2O_4$, magnetic $Ni^{2+}$ ion occupy A-sites, that is the tetrahedral sites, and magnetic $Cr^{3+}$ ions occupy B-sites, that is octahedral sites (O’Neill and Dollase 1994). The B-sites in the spinel structure form a network of tetrahedral sharing their corners. Consequently, magnetic $Cr^{3+}$ ($\mathrm{S}=3/2$) ions on the B-sites are geometrically frustrated with respect to the antiferromagnetic interaction between the nearest neighbors (Klemme and O'neill 2000; Lee et al. 2000, 2002; Bramwell, and Gingras 2001; Martinho et al. 2001; Ehrenberg et al. 2002). So even the Curie–Weiss temperature $\theta$ of $ZnCr_2O_4$ is −390 K indicating strong antiferromagnetic interaction, yet chromium spins remain in a paramagnetic phase down to about 12 K. Since $Cr^{3+}$ ion in cubic crystal is not a JT ion, that is, the ground state of $Cr^{3+}$ has no orbital degeneracy, $ZnCr_2O_4$ distorts to break the symmetry of the degenerated frustrated spin states by the spin-Peierls-like phase transition. Experimental results of low temperature X-ray diffraction (LTXRD), Xue et al. (2008) showed that below the transition temperature, the profile of (800) Bragg reflection splits into two peaks with lower angle peak having intensity twice that of the higher peak. The crystal distorted from cubic to tetragonal with $c < a$ with space group $I4_1/amd$. However, frustration driven magnetostructural coupling is not expected in the ferrimagnetic spinel $NiCr_2O_4.$ This is because $Ni^{2+}$–*O*–$Cr^{3+}$ interaction can be stronger than the frustrated interaction between the $Cr^{3+}$ ions (Reehuis et al. 2015; Suchomel et al. 2012). Furthermore, JT ion $Ni^{2+}$ can cause tetragonal distortions that should further alleviate frustration in the $Cr^{3+}$ sublattice. Low temperature structures and magnetic spin structures of $NiCr_2O_4$ have been studied by many authors (Suchomel et al. 2012; Ishibashi and Yasumi 2007; Tomiyasu and Kagomiya 2004). At 310 K co-operative JT distortion occurs with lowering the structural symmetry from cubic ($Fd\bar{3}m$) to tetragonal ($I4_1/amd$). Measurement of heat capacity, magnetic susceptibility and X-ray diffraction (XRD) show that the JT phase transition of $NiCr_2O_4$ compound occurs at $T_S= 310\;K$ with the elongated $NiO_4$ tetrahedron along the c-axis that is $c > a$ giving rise to the tetragonal structure with space group $Fd\bar{3}m$ to $I4_1/amd$ (Klemme and O'neill 2000; Klemme and van Miltenburg 2002; Ueno et al. 1999; Kino et al. 1972; Prince 1961). In the tetragonal phase the lattice constants ratio $c/a > 1$, in contrast with $ZnCr_2O_4$. Further distortion of tetragonal $NiCr_2O_4$ to orthorhombic phase occurs due to the ferrimagnetic ordering at $T_c=60K$. Bertaut and Dulac observed that both ferrimagnetic and antiferromagnetic ordering of $NiCr_2O_4$ simultaneously occur at $T_c=~65\; K$ (Bertaut and Dulac 1972, 1980). M.R. Suchomel et al. however, observed that at the ferrimagnetic transition of 65 K, the tetragonal (440) spectrum split into the orthorhombic (800) and (080) reflections by high-resolution synchrotron X-ray diffraction (Suchomel et al. 2012). Ishibashi and Yasumi (2007) reported another magnetic transition in $NiCr_2O_4$ at 31 K. Tomiyasu and Kagomiya also reported this temperature of $T_N = 31\; K$ corresponds to the ordering of the antiferromagnetic component of the magnetic structure of $NiCr_2O_4$ (Tomiyasu and Kagomiya 2004). Klemme and Miltenburg also observed anomaly in the specific heat at this temperature, but no changes in the average structure of $NiCr_2O_4$ have been observed at $T_N = 31\; K$ (Klemme and O'neill 2000). Among these three phase transitions, JT transition and the ferrimagnetic order accompany with crystal distortion from tetragonal to orthorhombic structure are well confirmed. The phase transition at 31 K is not yet well confirmed. This magneto-structural transition at 31 K should be necessary for further investigations. With the substitution of nonmagnetic $Zn^{2+}$ ion in addition to magnetic $Ni^{2+}$ ion in $NiCr_2O_4$, the magnetic interactions can be weakened and also the geometrical frustration can be enhanced. Study on $Ni_xZn_{1-x}Cr_2O_4$ system shows that the JT and magnetic phase transitions depend on the Ni concentration. It was observed by Kino et al. (1972) that when $x>0.6$ JT phase transition occurs at the higher temperature than magnetic phase transition. In $Ni_xZn_{1-x}Cr_2O_4$ system, $ZnCr_2O_4$ and $Ni_{0.5}Zn_{0.5}Cr_2O_4$ were already published in Ref. 8. $Ni_{0.5}Zn_{0.5}Cr_2O_4$ shows the transition temperatures at $T_S= 20\;K$ and $T_c= 16.5\;K$ respectively. In the present article, we have investigated the phase transitions of $Ni_xZn_{1-x}Cr_2O_4$ system with x = 0.6, 0.8 and 1 by measuring the low temperature X-ray diffraction. Not only the measurement of diffraction spectrum with Rietveld fitting but also the temperature dependence of full width at half maximum (FWHM), integrated intensity (I.I.) and the lattice spacing d of the powder specimen give the information about the phase transition. Especially we are interested in the magneto-elastic interactions and their effects with doping the nonmagnetic $Zn^{2+}$ ion in $NiCr_2O_4$. We would also like to investigate the phase transition at about 30 K in $NiCr_2O_4$. This phase transition is not yet well understood.

## Experiment

X-ray diffraction measurement for the powder specimens were performed using RINT 2500 system, Rigaku Company. The X-ray beam was generated by a rotating Cu anode. The whole profile of the reflection were measured with a step size of 0.20° and step-counting time of 1 s above and below the transition temperature for calculating the lattice constant by Rietveld refinements using “RIETAN-2000” (Izumi and Ikeda 2000). The temperature variation of the several short spectrum were taken at the higher angle. The FWHM and I.I. were obtained from the observed spectrum. Pseudo-Voight function is used to analyzes the short spectrum. The grain sizes of the particle are measured by the scanning electron microscope (SEM) FEI Company, Model inspect S50 with Tungsten filament.

## Sample preparations

The powder samples $Ni_xZn_{1-x}Cr_2O_4$ with x = 0.6, 0.8 and 1 were prepared from $ZnO\,(\ge\!99\;\%)$, $NiO\,(\ge\!99\;\%)$, $Cr_2O_3\,(\ge\!99\;\%)$ powders. The powders were mixed in appropriate proportions in an agate mortar under acetone for 3 h. The mixtures were then pre-sintered at 800 °C for 5 h. Resultant mixtures were reground and hand mixed for 3 h under acetone. The mixture were pressed into pellets and sintered at 1200 °C (Klemme and van Miltenburg 2004) for 5 h (increasing 10 °C/min). Powder X-ray diffraction (XRD) pattern at room temperature confirmed that the final product has a cubic-type spinel structure with single phase for 0.6 and 0.8 and tetragonal spinel structure with single phase for x = 1. Also XRD indicates no impurities for the samples x = 0.6 and 0.8. But the XRD indicated small impurity phases for the sample $NiCr_2O_4$. This is due to the raw materials NiO or $Cr_2O_3$ and Cu of the sample holder.

For the morphology test the samples $Ni_xZn_{1-x}Cr_2O_4$ with x = 0.6, 0.8 were prepared from the above mentioned powders. But the mixtures were pre-sintered at 1200 °C for 4 h with an increase in temperature 8 °C/min. Resultant mixtures were reground and hand mixed for 2 h under polyvinyl alcohol. The mixture was Pressed into pellets and sintered at 1500 °C for 4 h (increasing 8 °C/min) and they were polished to make the mirror surface.

## Results and discussions

### $NiCr_2O_4$

$$NiCr_2O_4$$ compound has a cubic spinel structure with the space group $$Fd\bar{3}m$$ to above 310 K. As described in introduction, it has been reported that $$NiCr_2O_4$$ shows the three phase transitions, that is at 310 K the JT distortion, at 65 K, the ferrimagnetic order and at 30 K antiferromagnetic ordering (Suchomel et al. 2012). To investigate these three phase transitions LTXRD was performed from 300 K down to 10 K. So it was not possible to observe the phase transition at 310 K. The whole profiles of reflection peaks were measured at 300, 100 and 15 K. The extra peak due to NiO or $$Cr_2$$$$O_3$$ and Cu are subtracted and then observed diffraction spectra were refined by the Rietveld refinements using “RIETAN-2000” software. The results of the Rietveld analysis at 300, 100 and 15 K are listed in Table 1(a–c). At 300 and 100 K the crystal structure is tetragonal with space group $$I4_1/amd$$. The refinement precisions are given by the value of S, 2.3491 and 1.1836 respectively. At 300 K, just below the phase transition temperature 310 K, the crystal structure transition should be transient. So Rietveld fitting gives rather worse S value than one at 100 K. These lattice parameters are completely identical with those obtained by Suchomel et al. (2012). At 15 K the crystal structure is orthorhombic with space group *Fddd* and the refinement precision is 1.29. The temperature variations of the several short spectra were measured between 10 and 300 K.

**Table 1:** Structural parameters obtained by “RIETAN-2000” of $NiCr_2O_4$ at 300, 100 and 15 K

Atom	Site	x	y	z	$B\ \AA ^2$
(a) 300 K Space group $I4_1/amd$					
a = b = $5.83367\pm 0.0193\;\AA$, c = $8.41297\pm 0.0278\;\AA$, Rwp = 20.13 %, Rp = 14.89 %, S = 2.3491					
Zn/Ni	4a	0.25	0.375	0.43	0.005
Cr	8d	0	0	0.23	0.002
O	16h	0.5108	0.2316	1.015	0.013
(b) 100 K Space group $I4_1/amd$					
a = b = $5.79207\pm 0.006\,\AA$, c = $8.54065\pm 0.091\,\AA$, Rwp = 6.94 %, Rp = 4.79 %, S = 1.1836					
Zn/Ni	4a	0.25	0.375	0.478	0.006
Cr	8d	0	0	0	0.003
O	16h	0.5093	0.22415	1.727	0.023
(c) 15 K Space group *Fddd*					
a = $8.17338\pm 0.009\,\AA$, b = $8.18914\pm 0.009\,\AA$, c = $8.56879 \pm 0.009\,\AA$, Rwp = 11.03 %, Rp = 8.29 %, S = 1.37					
Zn/Ni	4a	0.125	0.125	0.475	0.006
Cr	8d	0.5	0.5	0.5	0.003
O	16h	0.254	0.0274	0.862	0.011

**Fig. 1:**
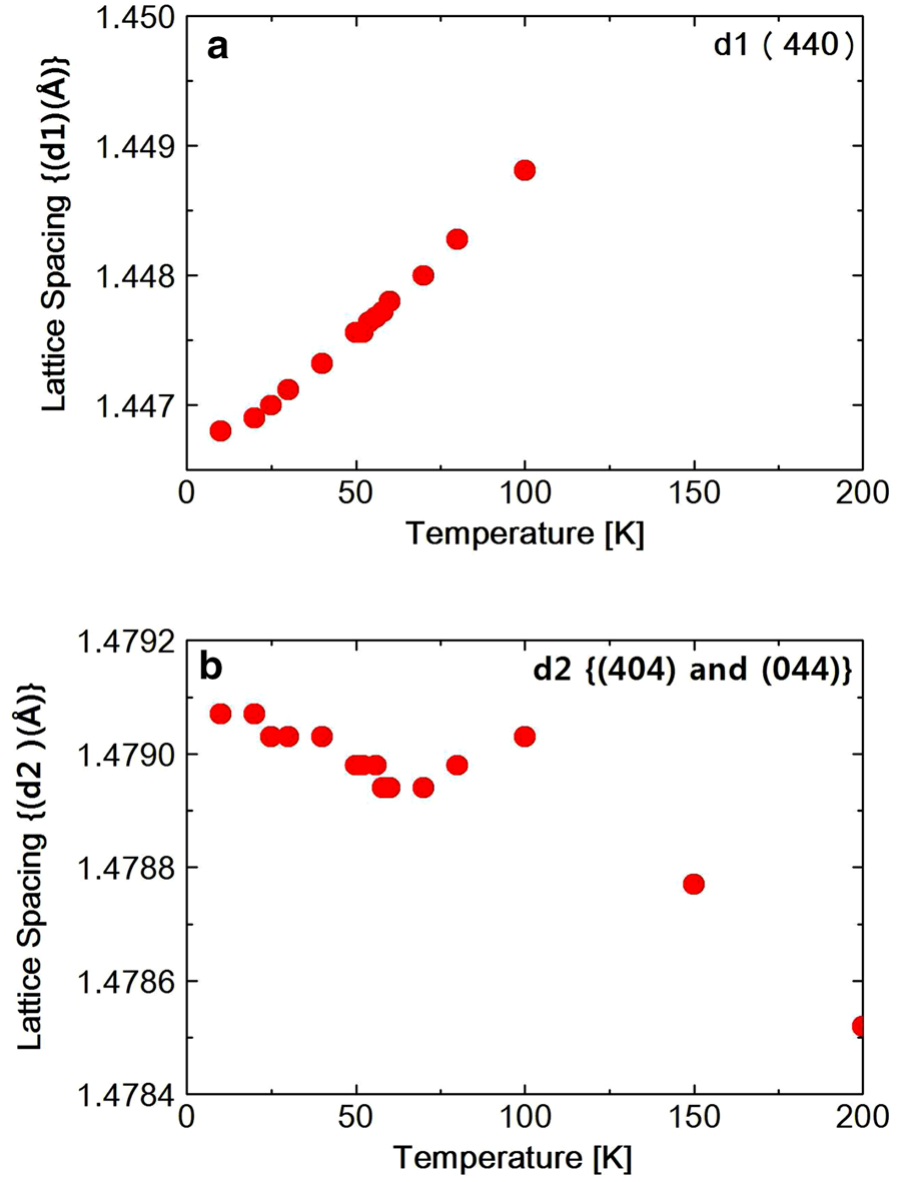
**a** Temperature dependence of the lattice spacing (*d*1) obtained from the (440) reflection in $NiCr_2O_4$. **b** Temperature dependence of the lattice spacing (*d*2) obtained from the reflection {(404) and (044)} in $NiCr_2O_4$

**Fig. 2:**
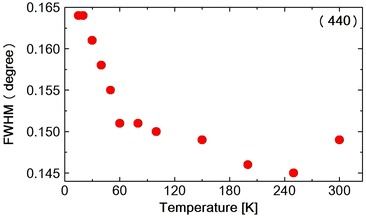
Temperature dependence of FWHM of (440) reflection in $NiCr_2O_4$

**Fig. 3:**
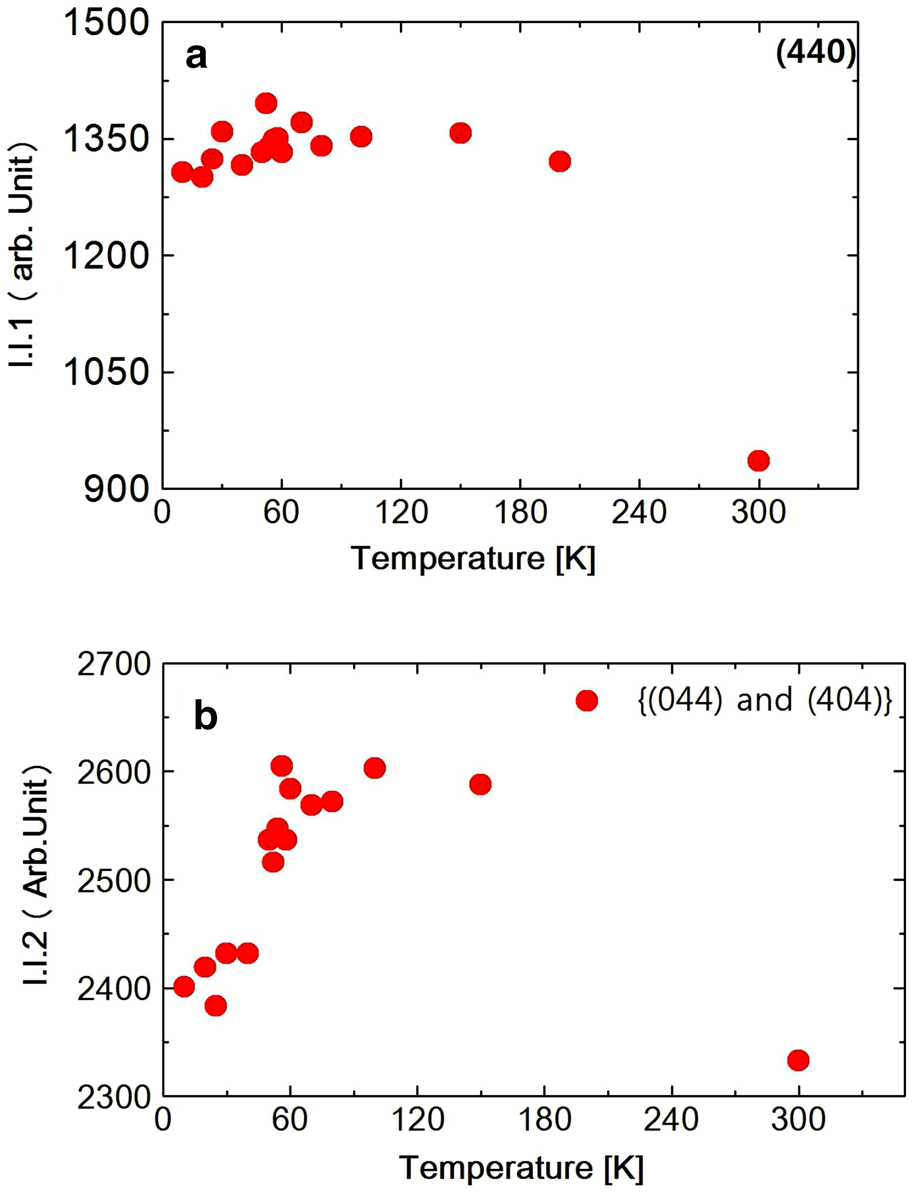
**a** Temperature dependence of I.I.1 of (440) reflection in $NiCr_2O_4$. **b** Temperature dependence of I.I.2 of {(404) and (044)} reflection in $NiCr_2O_4$

The diffraction intensity $I$ can be expressed by the following Debye–Waller equation Xue et al. (2008).

$$\begin{aligned} I\;=\;I_0\; exp\;(-2E_R\;sin^2\theta _B/3M\omega ^2) \end{aligned}$$

where $I_0$ is the scattered intensity from the rigid lattices, $\theta _B$ is the scattering angle, M is the mass of the atom and $\omega$ is the frequency of the oscillation and $E_R$ is the recoil-free energy. In this temperature range the spectrum was split into two peaks due to the tetragonal distortion. The spectrum was fitted to a Pseudo-Voigit function and the temperature dependence of the lattice spacing *d*1 (440) and *d*2 {(404) and (044)} were obtained as shown in Fig. 1a, b. At the ferrimagnetic transition at 65 K, M. R. Suchomel et al. observed the tetragonal (440) spectrum that split into orthorhombic (800) and (080) reflections as measured by high-resolution synchrotron X-ray powder diffraction. Unfortunately we did not observe the split of the tetragonal (440) reflection. The FWHM of tetragonal (440) and (044) reflection, however, increases abruptly below about 60 K as shown in Fig. 2, suggesting the structure distortion. The FWHM stopped to increase at 20 K. The I.I. also shows the abrupt decreasing below about 60 K, shown in Fig. 3a, b and also below about 25 K again slightly increased. These results of FWHM and I.I. suggest that at about 60 K, the structure distortion, might be occured from tetragonal to orthorhombic. As shown in Table 1c the Rietveld fitting gives the orthorhombic lattice constants at 15 K. At about 25 K the symmetry of the structure does not change but the lattice constant such as c-axis, changes its values due to the magneto-elastic interaction during the antiferromagnetic components orders. Tomiyasu and Kagomiya proposed the spin structure of $NiCr_2O_4$ from their neutron magnetic scattering and the magnetization measurements. Below $T_c$ = 75  K, the longitudinal component of the spins along the [100] direction order ferrimagnetically and below $T_N = 31\;K$, the transverse components of the spins along the [001] direction order antiferromagnetically. As shown in Fig. 1b, the lattice spacing *d*2 starts to increase below about 65 K and below about 30 K, it also increases slightly. These results should correspond to the increase in the magnetization below $T_c$ and also below $T_N$. The integrated intensity I.I.2 of {(404) and (044)} reflection spectrum as shown in Fig. 3b increases below 300 K due to the thermal effect shown in Debye–Waller equation. But it almost saturates below about 200 K. Then due to the softening of the lattice the I.I.2 of {(404) and (044)} reflection decreases below about 60 K. Below about 25 K it seems to be stopped to decrease. But the data around 25 K are rather scattering, so we will not discuss it anymore.

### $Ni_{0.8}Zn_{0.2}Cr_2O_4$

The size of $Ni_{0.8}Zn_{0.2}Cr_2O_4$ particles were checked by SEM. The distribution of particle size is large i.e., average particle size is about 1.85 μm. The smallest size of the particle is about 0.35 μm and the largest size of the particle is about 3.59 μm.

**Fig. 4:**
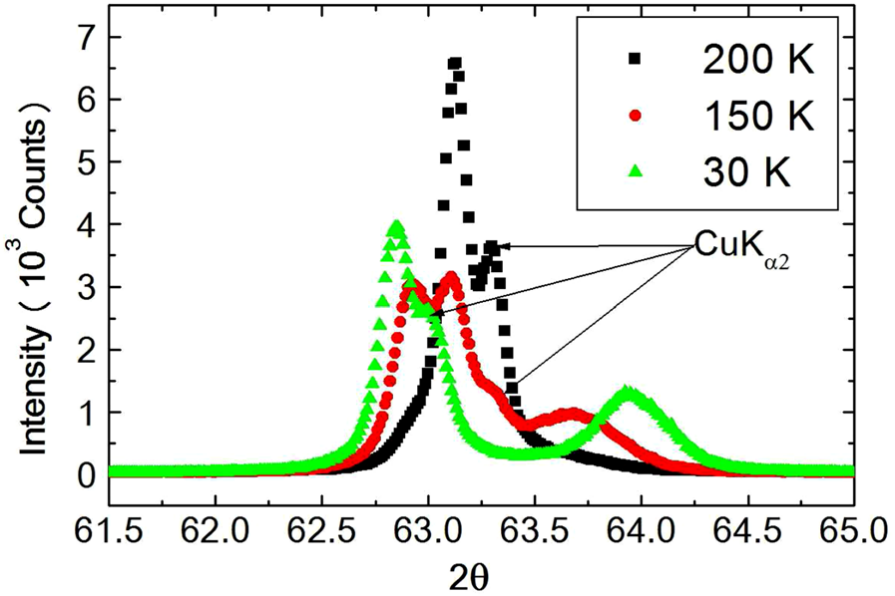
Profile of ($\theta -2\theta$) scans of (440) Bragg reflection of $Ni_{0.8}Zn_{0.2}Cr_2O_4$

**Fig. 5:**
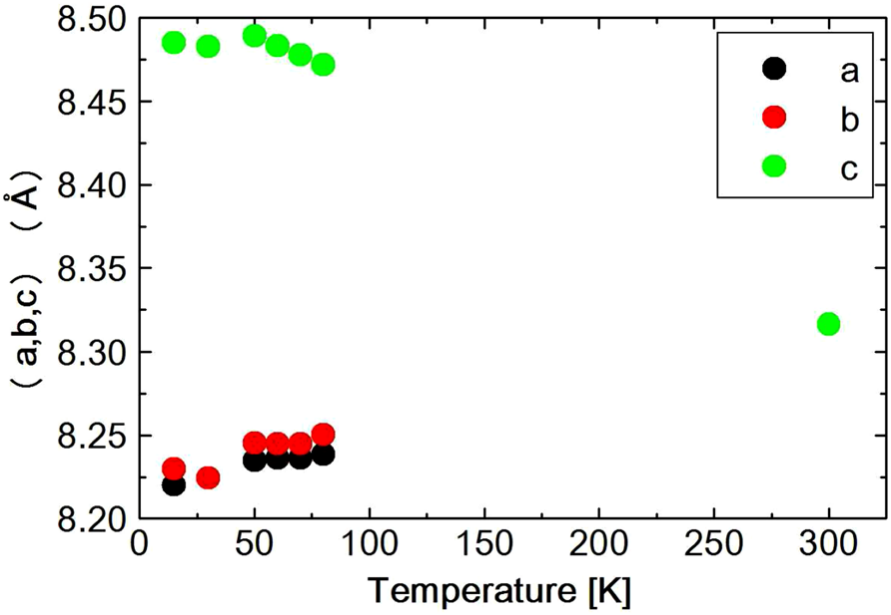
Temperature dependence of lattice parameters (*a*,* b*,* c*) in $Ni_{0.8}Zn_{0.2}Cr_2O_4$

**Fig. 6:**
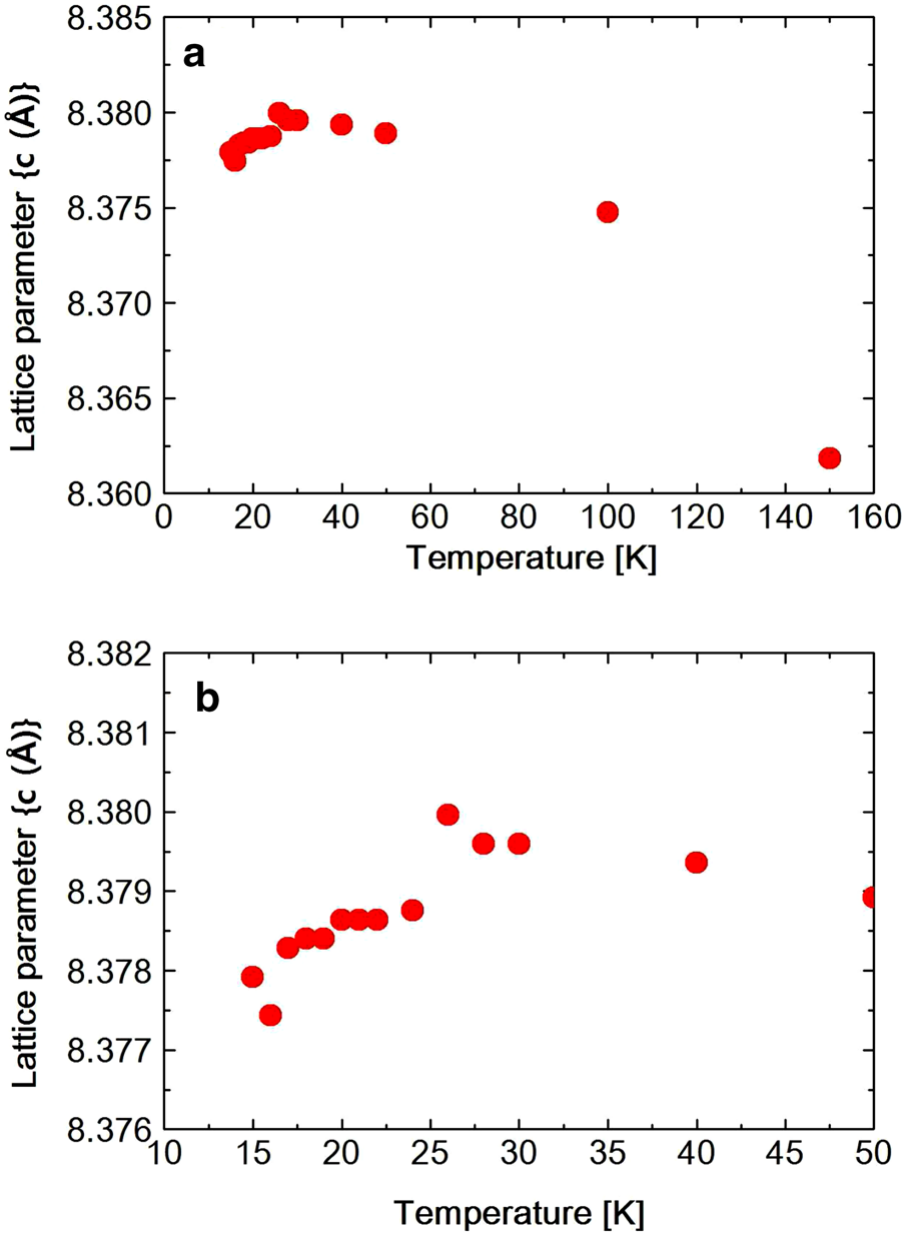
**a** Temperature dependence of lattice parameters (*c*) in $Ni_{0.8}Zn_{0.2}Cr_2O_4$. **b** Shows low temperature portion in expanded scale

**Fig. 7:**
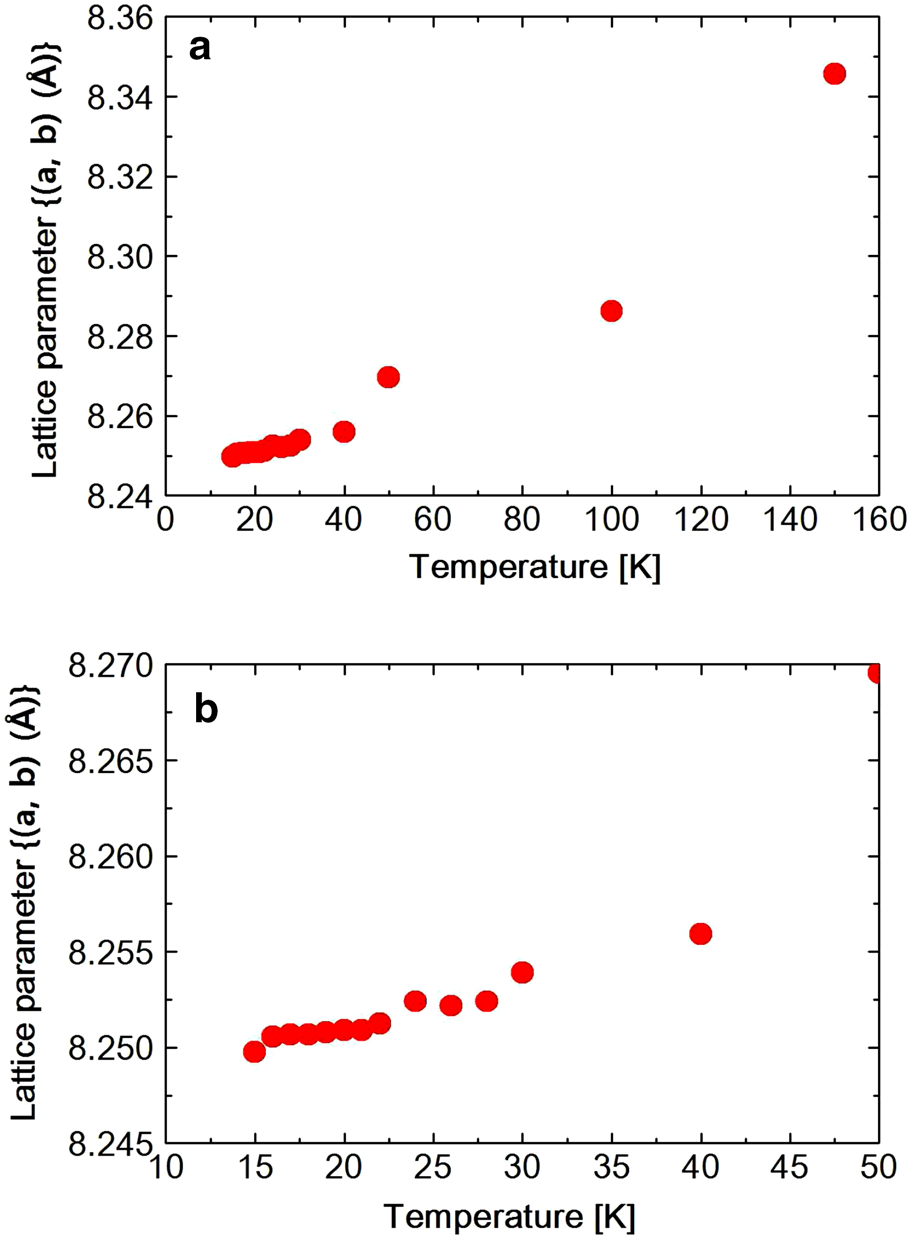
**a** Temperature dependence of lattice parameter $(<a, b>)$ in $Ni_{0.8}Zn_{0.2}Cr_2O_4$. **b** Shows low temperature portion in expanded scale

**Fig. 8:**
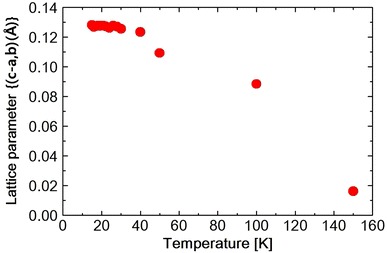
Temperature dependence of lattice parameter $(c{-} <a, b>)$ in $Ni_{0.8}Zn_{0.2}Cr_2O_4$

**Fig. 9:**
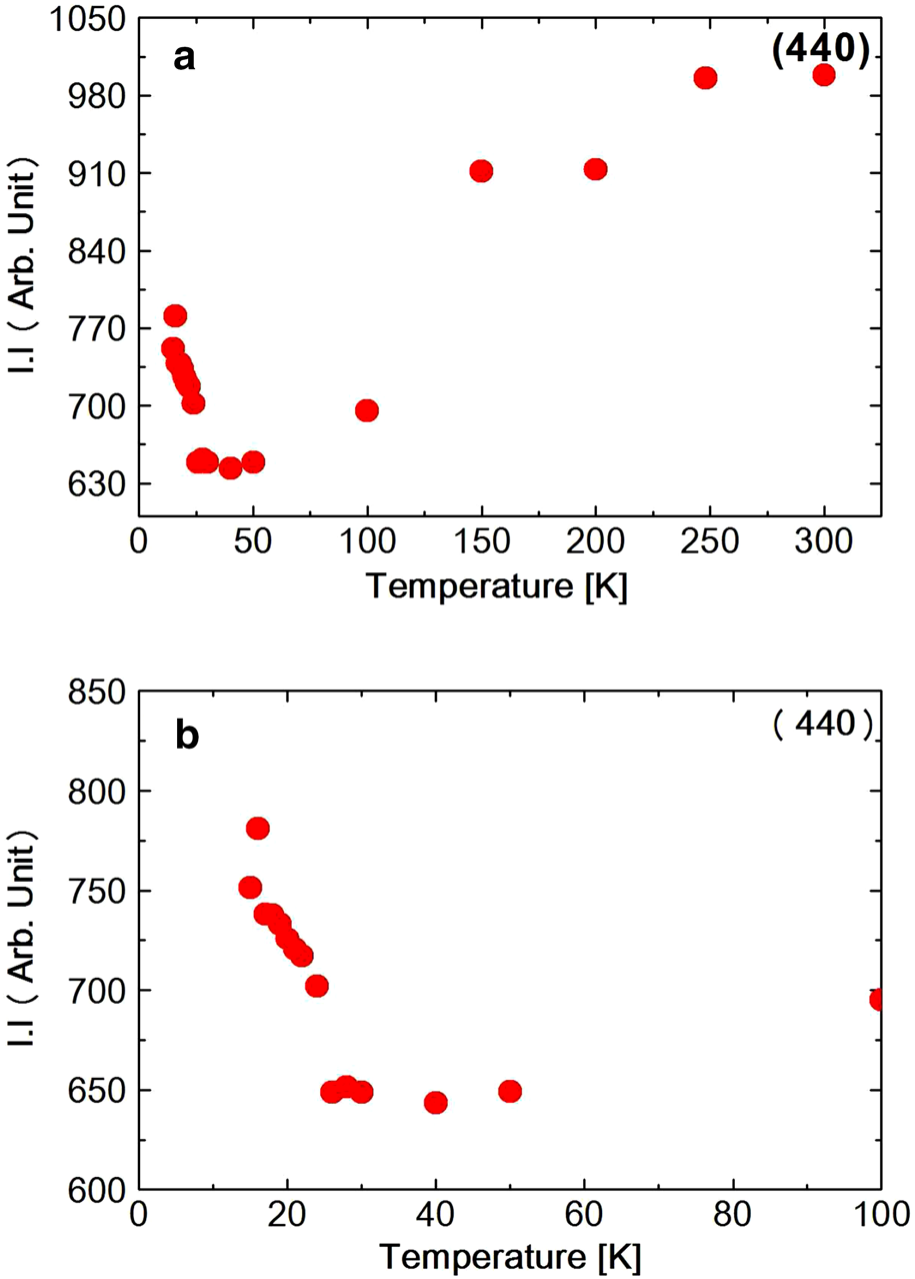
**a** Temperature dependence of I.I. obtain from the (440) reflection in $Ni_{0.8}Zn_{0.2}Cr_2O_4$. **b** Shows low temperature portion in expanded scale

**Fig. 10:**
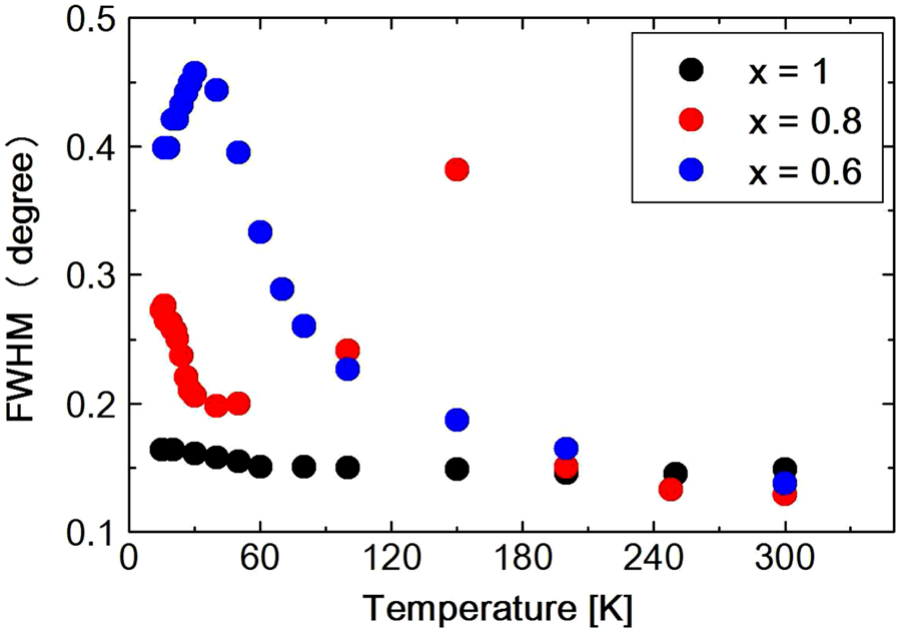
Temperature dependence of FWHM obtain from the (440) reflection in $Ni_xZn_{1-x}Cr_2O_4$

**Table 2:** Structural parameters obtained by “RIETAN-2000” $Ni_{0.8}Zn_{0.2}Cr_2O_4$ at 300, 80 K

Atom	Site	x	y	z	$B(\AA ^2)$
(a) 300 K Space group $Fd\bar{3}m$					
a = b = c = $8.31642\pm 0.001\,\AA$, Rwp = 10.61 %, Rp = 6.59 %, S = 1.2468					
Zn/Ni	4a	0.125	0.125	0.125	−
Cr	8d	0.5	0.5	0.5	−
O	16h	0.2602	0.2602	0.2602	−
(b) 80 K Space group *Fddd*					
a = $8.23861\pm 0.026\,\AA$, b = $8.25025\pm 0.026\,\AA$, c = $8.47176\pm 0.026\,\AA$, Rwp = 18.82 %, Rp = 14 %, S = 2.213					
Zn/Ni	8a	0.125	0.125	0.461	0.00584
Cr	8d	0.5	0.5	0.5	0.00499
O	32h	0.24958	0.27	0.872	0.01105

**Table 3:** Structural parameters obtained by “RIETAN-2000” $Ni_{0.8}Zn_{0.2}Cr_2O_4$ at 70, 60 K

Atom	Site	x	y	z	$B(\AA ^2)$
(a) 70 K Space group *Fddd*					
a = $8.23635\pm 0.022\,\AA$, b = $8.24477\pm 0.022\,\AA$, c = $8.4778\pm 0.022\,\AA$, Rwp = 17.93 %, Rp = 13.27 %, S = 2.107					
Zn/Ni	4a	0.125	0.125	0.414	0.00524
Cr	8d	0.5	0.5	0.356	0.00451
O	16h	0.25035	0.26948	0.769	0.00973
(b) 60 K Space group *Fddd*					
a = $8.23635\pm 0.022\,\AA$, b = $8.24477\pm 0.022\,\AA$, c = $8.48328\pm 0.023\,\AA$, Rwp = 17.86 %, Rp = 13.37 %, S = 2.097					
Zn/Ni	8a	0.125	0.125	0.361	0.00457
Cr	8d	0.5	0.5	0.332	0.00421
O	32h	0.25035	0.26948	0.727	0.0092

**Table 4:** Structural parameters obtained by “RIETAN-2000” $Ni_{0.8}Zn_{0.2}Cr_2O_4$ at 50, 30 and 15 K

Atom	Site	x	y	z	$B(\AA ^2)$
(a) 50 K Space group *Fddd*					
a = $8.23506\pm 0.023\, \AA$, b = $8.24518\pm 0.023\, \AA$, c = $8.48909\pm 0.023\, \AA$, Rwp = 17.51 %, Rp = 13.07 %, S = 2.0588					
Zn/Ni	4a	0.125	0.125	0.334	0.00424
Cr	8d	0.5	0.5	0.296	0.00375
O	16h	0.25123	0.27039	0.665	0.00842
(b) 30 K Space group *Fddd*					
a = $8.22436\pm 0.005\,\AA$, b = $8.22444\pm 0.004\, \AA$, c = $8.48298\pm 0.005\, \AA$, Rwp = 17.86 %, Rp = 13.37 %, S = 2.097					
Zn/Ni	4a	0.125	0.125	0.474	0.006
Cr	8d	0.5	0.5	0.348	0.00441
O	16h	0.26161	0.26706	0.835	0.01057
(c) 15 K Space group *Fddd*					
a = $8.22003\pm 0.005\, \AA$, b = $8.22974\pm 0.005\, \AA$, c = $8.48524\pm 0.005\, \AA$, Rwp = 16.53 %, Rp = 11.13 %, S = 2.1605					
Zn/Ni	4a	0.125	0.125	0.516	0.00654
Cr	8d	0.5	0.5	0.387	0.00449
O	16h	0.25157	0.026654	0.859	0.01088

#### Crystal structure

As discussed in the previous section, three phase transitions in $NiCr_2O_4$ are almost confirmed, though the third phase transition at about 30 K is still not well understood. With doping the nonmagnetic $Zn^{2+}$ ion in place of the magnetic $Ni^{2+}$ ion in the parent compound $NiCr_2O_4$, these three phase transition temperatures can be expected to be lower temperatures. To investigate the crystal structure of the compound $Ni_{0.8}Zn_{0.2}Cr_2O_4$ whole profiles of reflection peaks were measured at several temperatures 300, 80, 70, 60, 50, 30 and 15 K and refined by the Rietveld method. Structural parameters obtained by “RIETAN-2000” at 300, 80, 70, 60, 50, 30 and 15 K are listed in Tables 2, 3 and 4 respectively. At room temperatures, the prepared $Ni_{0.8}Zn_{0.2}Cr_2O_4$ spinel compounds are rather homogeneous compound in the space group $Fd\bar{3}m$ with lattice parameter $8.31642\;\pm \;0.001\;\AA$ and the refinement precision is given by the value of S = 1.2468. The overall quality of the fitting is fairly good. Profiles of $(\theta -2\theta )$ scans of (440) Bragg reflection at 200 K $(T>T_S)$, 150 K $(T \le T_S)$ and 30 K $(T<T_S)$ are shown in Fig. 4. These profiles contain $CuK_{\alpha 2}$ in the right shoulder of the peaks. At 150 K the profile of (440) already split into two peaks. This phase transition undergoes by JT interaction and also spin-Peierls-like interaction and as shown in Table 2, the symmetry of the crystal lowers from $Fd\bar{3}m$ to *Fddd*.

The lattice constants obtained by the Rietveld refinement which are listed in Tables 2, 3 and 4 are plotted against temperature in Fig. 5. As shown in the table or in the figure the lattice constants a and b take nearly same value at 30 K. This, however, should be accidental. Because at 15 K, a and b take different values again. The $$Ni_{0.8}Zn_{0.2}Cr_2O_4$$ (440) reflection is measured at various temperatures between 15 and 300 K. The spectrum was fitted to a Pseudo-Voigit function and the temperature dependence of the lattice constants is obtained and shown in Figs. 6, 7 and 8 and also I.I. is shown in Fig. 9. The FWHM, of the spectrum, together with that of the $$NiCr_2O_4$$ (440) reflection and $$Ni_{0.6}Zn_{0.4}Cr_2O_4$$ (440) reflection are shown in Fig. 10. Here for $$Ni_{0.8}Zn_{0.2}Cr_2O_4$$ compound the measurement was carried out twice. The two results are almost same, though there is a small scattering. At 300 K, the FWHM of $$Ni_{0.8}Zn_{0.2}Cr_2O_4$$ compound is much smaller than that of $$NiCr_2O_4$$. This is because the structural phase transition of $$NiCr_2O_4$$ is just at higher temperature of 310 K. But this result suggests that the homogeneity of the $$Ni_{0.8}Zn_{0.2}Cr_2O_4$$ compound is rather good. At 250 K, the FWHM of $$Ni_{0.8}Zn_{0.2}Cr_2O_4$$ is almost same as that of $$NiCr_2O_4$$. It also supports the good homogeneity of $$Ni_{0.8}Zn_{0.2}Cr_2O_4$$ compound. With decreasing the temperature the FWHM of $$Ni_{0.8}Zn_{0.2}Cr_2O_4$$ starts to increase and reach the sharp peak at 160 K, then decreases rather rapidly down to about 40 K and finally again starts to increase below about 30 K. The result of the Rietveld fitting shows that the low temperature structures can be orthorhombic. In the distorted phase the S value is much larger than 2, suggesting the worse fitting of Rietveld. We have to discuss the origin of this worse Rietveld fitting. Doping the $$Zn^{2+}$$ ion in place of $$Ni^{2+}$$, results in the mixed compound of $$NiCr_2O_4$$ and $$ZnCr_2O_4$$. In the mixed compound, the local distortion of $$NiCr_2O_4$$$$(c/a>1)$$ and $$ZnCr_2O_4$$$$(c/a<1)$$ separately occurs so as to minimize the crystal distortion energy. Then as M. Kataoka and J. Kanamori has already mentioned in their theoretical work on $$Cu_{1-x}Ni_xCr_2O_4$$ system Kataoka and Kanamori (1972), the elongated c-axis of $$NiCr_2O_4$$ will align along one of a-axes of $$ZnCr_2O_4$$, say a- or b-axis which are also elongated in $$ZnCr_2O_4$$. If the alignment of c-axis prefers to a particular axis, say a-axis, a and b axes are not equal, so the result is orthorhombic. When the distorted $$NiCr_2O_4$$ with $$c/a>1$$ and the distorted $$ZnCr_2O_4$$ with $$c/a<1$$ are mixed in $$Ni_{0.8}Zn_{0.2}Cr_2O_4$$ compound below the structural transition temperature, the FWHM can be expected to increase. In Fig. 8$$(c{-}< a,\;b>)$$ vs. T is plotted. Here $$<a,\;b>$$ is the average values of lattice constants a and b obtained from the reflection (440) measurement. The figure shows that the structure transition seems to be at 160 K which corresponds to the peak temperature of FWHM. With decreasing temperature, $$(c{-} <a, b >)$$ increases and is goes to saturate at low temperatures, while the FWHM decreases quickly. This result can be understood as follows. At the transition temperatures, the c-axis of local $$NiCr_2O_4$$ structure aligns rather randomly and with lowering the temperature the alignment becomes more stable to one of a-axes. In present Rietveld fitting, the S value is rather large in the distorted phase. The FWHM in $$Ni_{0.8}Zn_{0.2}Cr_2O_4$$ starts to increase rather sharply below about 30 K (Fig. 10). This behavior is similar to one in $$NiCr_2O_4$$ below about 60 K which corresponds to the ferrimagnetic ordering temperature. In $$NiCr_2O_4$$, the ferrimagnetic transition accompany with the crystal distortion from the tetragonal symmetry to the orthorhombic. $$Ni_{0.8}Zn_{0.2}Cr_2O_4$$ is however, orthorhombic structure above the ferrimagnetic transition. So only the lattice constants can be changed with spin order. As shown in Fig. 7, the lattice constants a or b do not show the drastic change at the ferrimagnetic order, but the c axis as shown in Fig. 6 shows the sudden drop at the ordering temperature. The I.I. of $$Ni_{0.8}Zn_{0.2}Cr_2O_4$$ in Fig. 9 also shows the sudden increase at about 25 K. The Rietveld fitting S value at 15 K shows a large value of 2.15. But when we analyze the spectrum measured at 15 K by using the model of *Fddd* and $$I4_1/amd$$ symmetry mixing, S value is 1.81, not so small but much smaller than the S = 2.15 obtained assuming only $$I4_1/amd$$ structure model for the same spectrum at 15 K. In the distorted phase between 30 and 160 K, it is better to analyze the spectrum by using the model of *Fddd* and a slight mixing of $$I4_1/amd$$. At about 16 K, the I.I. also shows the abrupt drop and the lattice constant c and a, b also drop at this temperature and the FWHM almost saturate below this temperature. From the temperature variation of I.I. of $$NiCr_2O_4$$ and that of $$Ni_{0.8}Zn_{0.2}Cr_2O_4$$, it is found that these two figures are very similar to each other. At about 150 K $$(Ni_{0.8}Zn_{0.2}Cr_2O_4)$$ and 60 K $$(NiCr_2O_4)$$, I.I. decrease very sharply and at 25 K $$(Ni_{0.8}Zn_{0.2}Cr_2O_4)$$ and 20 K $$(NiCr_2O_4)$$, I.I. abruptly increase. The phase transition of $$NiCr_2O_4$$ at 60 K is the ferrimagnetic order accompanied with the structural change from tetragonal to orthorhombic phase. So, the structural phase transition is due to the magneto-elastic interaction. The structural phase transition of $$Ni_{0.8}Zn_{0.2}Cr_2O_4$$ at 160 K may also be due to the magneto-elastic coupling, that is, the collaboration of JT interaction due to the $$Ni^{2+}$$ ion and spin-Peierls-like interaction due to the $$Cr^{2+}$$ ion. The phase transition of $$Ni_{0.8}Zn_{0.2}Cr_2O_4$$ at 25 K is the ferrimagnetic transition, but it also accompany with the structure change. But it already has the orthorhombic symmetry. So it also does not change the symmetry and just changes the lattice constants, similar to the phase transition which occurs at 30 K in $$NiCr_2O_4$$. In Ni$$Cr_2$$$$O_4$$, the ferrimagnetic transition occurs at 60 K, but it accompany with the crystal symmetry change. In $$NiCr_2O_4$$ the antiferromagnetic transition without the crystal symmetry change occurs at 30 K. In $$Ni_{0.8}Zn_{0.2}Cr_2O_4$$ another phase transition also occurs at about 16 K which should be antiferromagnetic transition similar to one at 30 K in $$NiCr_2O_4$$.

### $Ni_{0.6}Zn_{0.4}Cr_2O_4$

The particle sizes of the $$Ni_{0.6}Zn_{0.4}Cr_2O_4$$ were also checked by SEM. The distribution of particle size is large. The average particle size is about 1.92 μm. The smallest size of the particle is about 0.78 μm and the largest size of the particle is 3.46 μm. The ionic radius of $$Zn^{2+}$$ is about $$0.75\;\AA$$ which is larger than ionic radius of $$Ni^{2+}$$$$(0.70\;\AA )$$. So the average size of the particle for the sample $$Ni_{0.6}Zn_{0.4}Cr_2O_4$$ will be larger than the $$Ni_{0.8}Zn_{0.2}Cr_2O_4$$. Experimental results show the same as were expected.

**Fig. 11:**
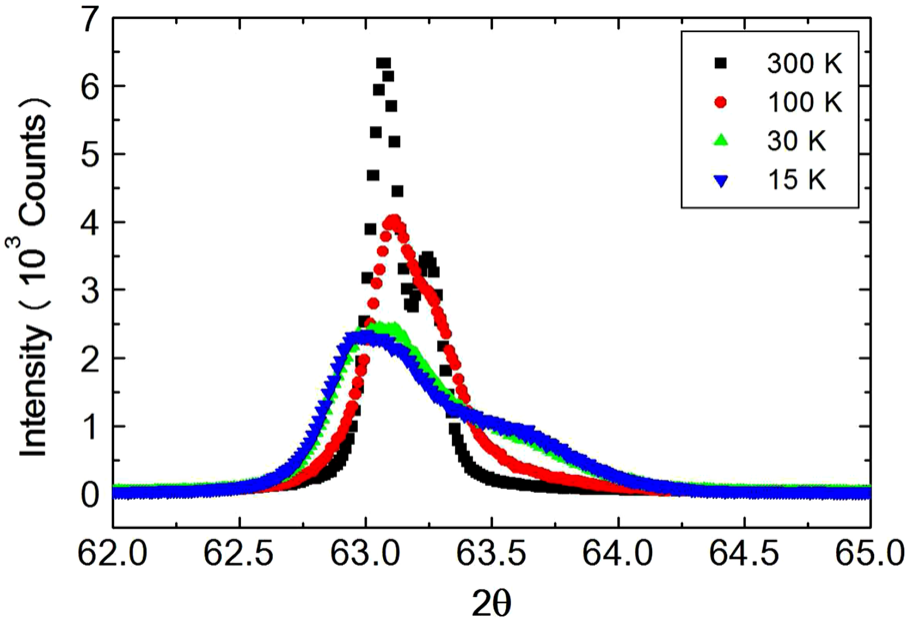
($\theta -2\theta$) scans of (440) Bragg reflection of $Ni_{0.6}Zn_{0.4}Cr_2O_4$

X-ray diffraction spectrum does not show any clear split due to the crystal distortion. Profiles of $(\theta - 2\theta )$ scans of (440) Bragg reflection at 300 K $(T>T_S)$, 100 K $(T>T_S)$, 30 K $(T\cong T_S)$ and 15 K $(T<T_S)$ are shown in Fig. 11. These profiles contain $CuK_{\alpha 2}$ in the right shoulder of the peaks.

**Fig. 12:**
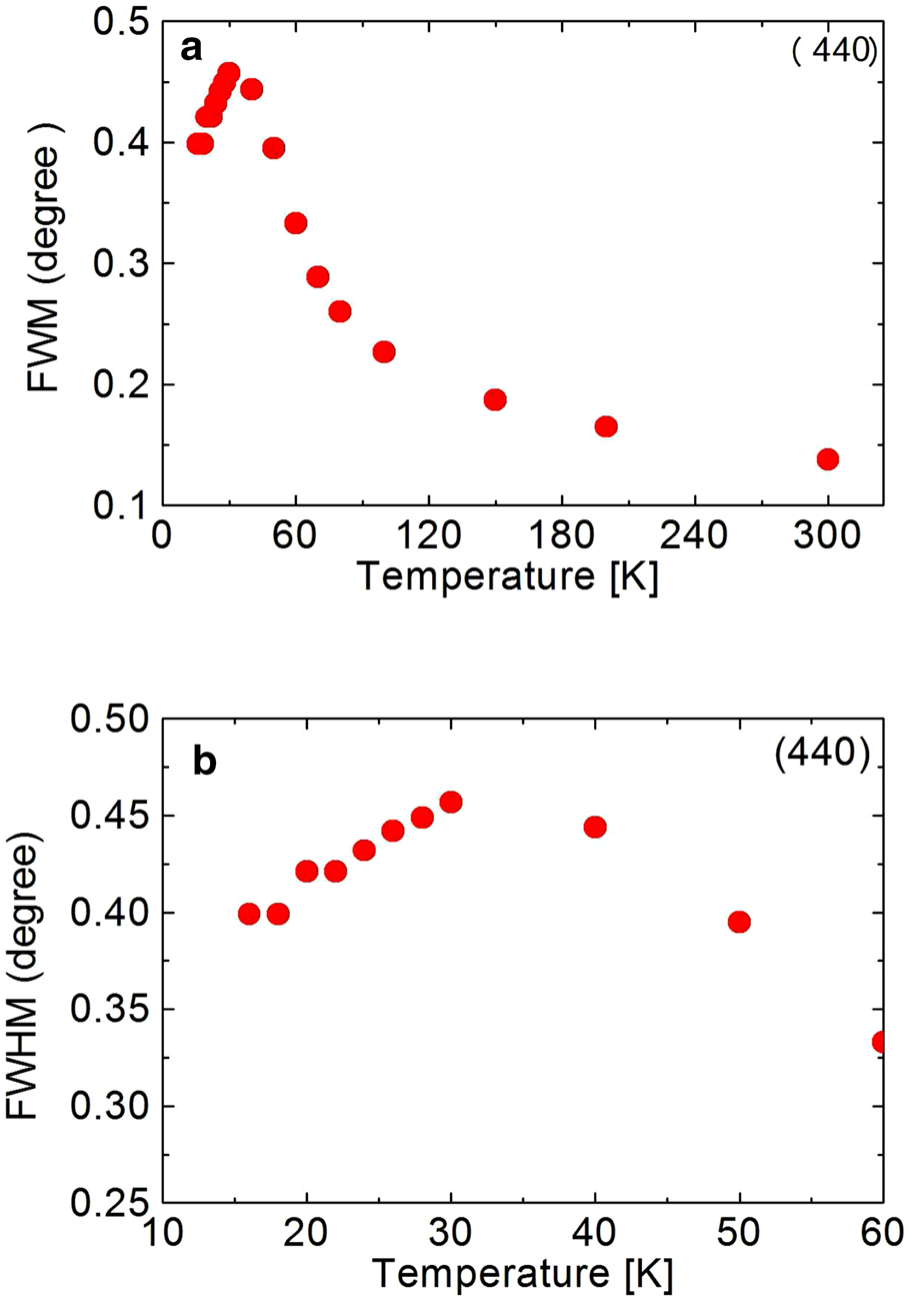
**a** Temperature dependence of FWHM obtained from the (440) reflection in $Ni_{0.6}Zn_{0.4}Cr_2O_4$. **b** Low temperature portion in expanded scale

**Fig. 13:**
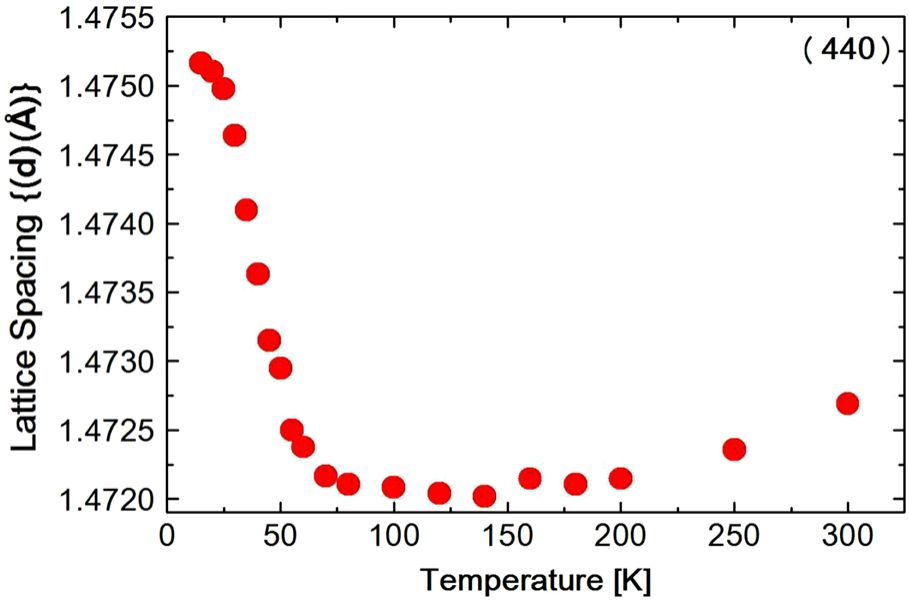
Temperature dependence of d value obtain from the (440) reflection in $Ni_{0.6}Zn_{0.4}Cr_2O_4$

**Fig. 14:**
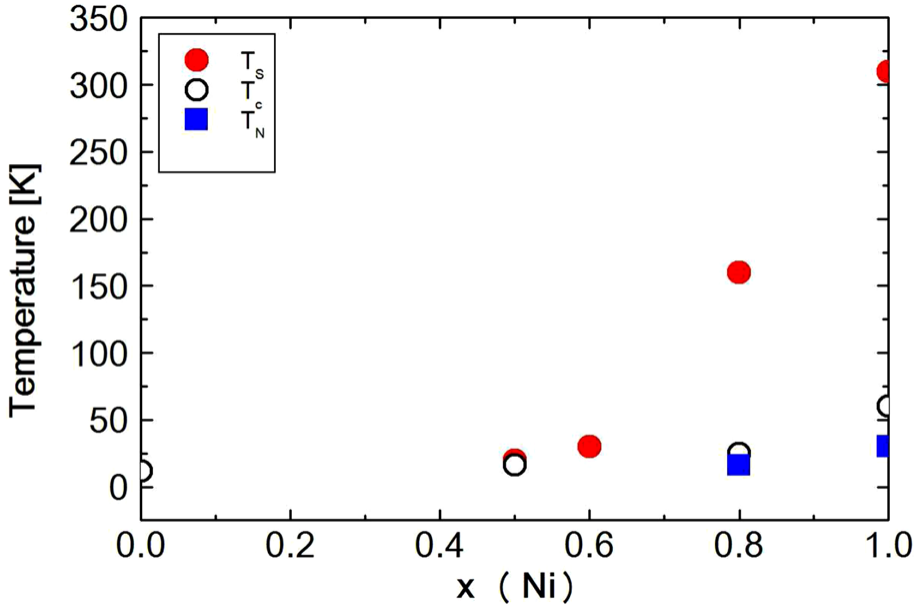
The phase diagram of $Ni_xZn_{1-x}Cr_2O_4$, with temperature dependence of Ni concentrations

**Fig. 15:**
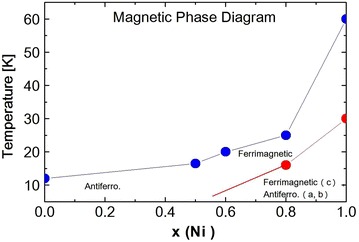
The magnetic phase of $Ni_xZn_{1-x}Cr_2O_4$, with temperature dependence of Ni concentrations

At temperature 30 K $(T\cong T_S)$ the peak is rather broad. But FWHM shows the maximum at about 30 K, as shown in Fig. 12. In the case of $Ni_{0.8}Zn_{0.2}Cr_2O_4$, FWHM shows the very sharp peak. This peak may be attributed to the crystal structure transition. The broad peak at about 30 K in $Ni_{0.6}Zn_{0.4}Cr_2O_4$ may also be due to the structural phase transition. The crystal structure below the structural phase transition temperature $T_S$ can be the tetragonal, $I4_1/amd$ or the orthorhombic, *Fddd*, but not yet determined. The most possible structure should be the mixed structure of $I4_1/amd$ and *Fddd* which can produce the broad peak of FWHM. The temperature dependence of the FWHM also shows another anomaly at about 20 K. This can be the magnetic phase transition such as the paramagnetic to ferrimagnetic state. The third phase transition which can be observed in $NiCr_2O_4$ and in $Ni_{0.6}Zn_{0.4}Cr_2O_4$ cannot be seen in the present measurements down to 12 K in $Ni_{0.6}Zn_{0.4}Cr_2O_4$. On the other hand, the lattice spacing d value for (440) reflection increases below about 80 K with decreasing temperature as shown in Fig. 13. This result must correspond to the FWHM results, that is, showing the change in crystal structure at about 30 K. It can be understood as follows. The reflection of (440) can split into two reflections (440) and {(404), (044)}. This tetragonal structure can be $c/a > 1$, similar to $Ni_{0.8}Zn_{0.2}Cr_2O_4$ compound. The integrated intensity of {(404), (044)} reflection can be twice that of (440) reflection. The sample $Ni_{0.6}Zn_{0.4}Cr_2O_4$ is rather inhomogeneous. So the reflection peaks of (440) and {(404), (044)} are broad. Even in the tetragonal phase the reflection peak shows one broad peak as shown in Fig. 11. The behavior of this broad peak can be determined by the large intensity of {(404), (044)} reflections. The d value obtained from the (440) reflections starts to increase below about 80 K with decreasing temperature. In the case of $Ni_{0.8}Zn_{0.2}Cr_2O_4$ compound, the I.I. also shows the anomaly at the phase transition of $T_S$ and $T_c$. But in $Ni_{0.6}Zn_{0.4}Cr_2O_4$ compound, the I.I. does not show clear anomaly at these phase transition temperatures. This result can be understood by the inhomogeneity of the compound. The phase diagram of $Ni_xZn_{1-x}Cr_2O_4$ shown in Fig. 14. The magnetic phase diagram part is also shown in Fig. 15. c axis is along (001), a and b are perpendicular direction to (001). In ferrimagnetic spin structure, the spins align along (001) direction, but slightly canted and the perpendicular components of c axis order antiferromagnetically.

## Conclusions

Low temperature X-ray diffraction studies were done for x = 1, 0.8 and 0.6. It has been observed that in $NiCr_2O_4$, ferrimagnetic transition $T_N$ occurs at 60 K and antiferromagnetic transition occurs at 30 K. In $Ni_{0.8}Zn_{0.2}Cr_2O_4$ JT transition occurs at temperature 150 K and another phase transition occurs at 30 K. The lattice parameters obtained by the Rietveld method reveal that magnetic ordering is accompanied with $a\ne b$. In $Ni_{0.6}Zn_{0.4}Cr_2O_4$ the intensity of the spectrum started to fall at 100 K. The FWHM increases as the temperature decreases down to 30 K below which it started to decrease, indicating the crystal phase transition. The FWHM also shows another phase transition from JT to ferrimagnetic at temperature 20 K. The morphology test of the samples $Ni_{0.8}Zn_{0.2}Cr_2O_4$ and $Ni_{0.6}Zn_{0.4}Cr_2O_4$ shows that as the $Zn^{2+}$ concentration increases the average particle size of the sample increases.
